# Quality of Life Outcomes Following Aortofemoral and Iliofemoral Bypass Surgery in Patients With Peripheral Arterial Disease: A Two-Year Follow-Up Study

**DOI:** 10.7759/cureus.61598

**Published:** 2024-06-03

**Authors:** Saeed Alqahtani, Fahad Aljaber, Bander Alharbi, Riyadh Masoud

**Affiliations:** 1 Vascular Surgery, Charité – Universitätsmedizin Berlin, Berlin, DEU; 2 Vascular Surgery, Prince Sultan Military Medical City, Riyadh, SAU; 3 Vascular Surgery, Samsung Medical Center, Seoul, KOR

**Keywords:** aortofemoral bypass, surgical outcomes, quality of life, iliofemoral bypass, peripheral arterial disease

## Abstract

Introduction: Peripheral arterial disease is a circulatory disorder characterized by reduced blood flow to the extremities, predominantly affecting the lower limbs. This study aims to evaluate the impact of aortofemoral and iliofemoral bypass surgeries on patients' quality of life two years post operation and identify predictors of quality-of-life improvements.

Methods: This cross-sectional study included adult patients with aortoiliac disease who underwent bypass surgery (aortofemoral or iliofemoral) at East Jeddah General Hospital from January 2020 to December 2022. Quality of life was assessed using the Arabic version of the Short Form Health Survey 12 (SF-12) preoperatively and two years postoperatively. Data on sociodemographic factors (age, sex, education, income) and medical factors (smoking, BMI, comorbidities) were collected. Statistical analyses included descriptive statistics, t-tests, one-way ANOVA, and regression analyses using IBM SPSS version 25.0 (IBM Corp., Armonk, NY).

Results: The study included 275 patients. Significant improvements in both physical and mental SF-12 scores were observed postoperatively across all patient groups (P < 0.001). Older age, unemployment, and lower income were associated with lower SF-12 scores. Males had higher postoperative mental scores (P = 0.036). Higher BMI and smoking pack-years negatively correlated with SF-12 scores. Patients with comorbidities had significantly lower preoperative and postoperative SF-12 scores (P < 0.05) but showed significant improvements postoperatively (P < 0.001).

Conclusion: Aortofemoral and iliofemoral bypass surgeries significantly improve the quality of life in peripheral arterial disease patients two years post operation. Key predictors of lower quality of life include older age, unemployment, lower income, high BMI, smoking, and comorbidities. Targeted interventions, such as smoking cessation programs, weight management, and comprehensive medical care, are essential for optimizing postoperative outcomes and enhancing patients' physical and mental well-being.

## Introduction

Peripheral arterial disease is a circulatory disorder characterized by reduced blood flow to the extremities, predominantly affecting the lower limbs. It is estimated that approximately 200 million adults worldwide are affected by peripheral arterial disease, with its incidence rising notably in individuals over the age of 70 years [1,2]. The disease typically presents with lower limb pain on exertion and at rest in severe cases. Atherosclerotic plaques, which narrow arterial flow lumens, are the primary pathological mechanism underlying peripheral arterial disease [3,4].

Numerous risk factors contribute to the development and progression of peripheral arterial disease, including race, ethnicity, uncontrolled diabetes, smoking, obesity, hypertension, dyslipidemia, increased age, family history of peripheral arterial disease, and elevated levels of homocysteine [5,6]. For instance, individuals with a history of heavy smoking or poorly controlled diabetes exhibit a significantly heightened risk of peripheral arterial disease, often associated with increased disease severity and elevated risks of limb amputation [7,8].

Lifestyle modifications, encompassing physical exercise and dietary adjustments, play pivotal roles in both preventing and managing peripheral arterial disease [7]. However, implementing these modifications can be challenging, particularly as peripheral arterial disease limits patients' exercise tolerance, exacerbating the underlying pathological mechanisms. Pharmacological interventions, such as cilostazol and supervised exercise programs, have shown promise in improving functional performance and mitigating symptoms [9].

When peripheral arterial disease progresses beyond the point where medications and lifestyle modifications provide substantial benefit, surgical interventions become necessary [10]. Despite advancements in endovascular techniques, surgical procedures remain fundamental in peripheral arterial disease management, addressing challenges such as occlusive disease patterns, patient fitness for anesthesia, extent of tissue loss, and anatomical considerations. Endovascular surgery, compared to open approaches, has demonstrated favorable outcomes in terms of complication rates, hospitalization duration, and long-term mortality [11].

Among the most commonly employed surgical interventions for peripheral arterial disease are bypass procedures, which offer versatility in treating arterial occlusions ranging from the aorta to the foot. Bypass surgeries have shown favorable long-term patency rates and improvements in quality-of-life measures, as evidenced by studies utilizing the Short Form-36 (SF-36) questionnaire [12]. However, there is a paucity of research assessing the long-term impact of bypass surgery on patients' quality of life two years post operation. Therefore, our current study aims to elucidate the impact of aortofemoral and iliofemoral bypass surgeries on patients' quality of life two years post operation. Additionally, we seek to identify sociodemographic and medical predictors associated with quality-of-life improvements in these patient populations.

## Materials and methods

Study design

This study employed a cross-sectional design to assess the impact of lower limb revascularization surgery on patients' quality of life. Data were collected at two time points: preoperatively and two years postoperatively.

Study participants

The study included adult patients diagnosed with aortoiliac disease who underwent bypass surgery (aortofemoral or iliofemoral) at East Jeddah General Hospital between January 2020 and December 2022. Inclusion criteria were patients aged 18 years and older with documented aortoiliac disease requiring surgical intervention. Exclusion criteria included patients with non-aortoiliac peripheral arterial disease, those unable to complete the Short Form Health Survey 12 (SF-12) questionnaire, and patients who underwent non-bypass revascularization procedures.

Surgical procedures

Aortofemoral and iliofemoral bypass surgeries were performed by a team of experienced vascular surgeons. The choice of graft (synthetic or autologous vein) and specific surgical technique (end-to-end anastomosis, end-to-side anastomosis) was based on individual patient anatomy and disease severity. Intraoperative management adhered to standard protocols, including anticoagulation, anesthesia, and postoperative care.

Data collection

Patients' quality of life was assessed using the validated Arabic version of the SF-12. The SF-12 comprises physical and mental components and consists of 12 Likert scale-based questions [13]. Patients completed the SF-12 questionnaire during preoperative consultations and at a follow-up visit two years postoperatively. Additional data collected preoperatively included patients' symptoms, ankle-brachial pressure index (ABPI), and a comprehensive sociodemographic and medical history (age, sex, education, income, smoking status, BMI, and comorbidities). Data were entered into a secure database and anonymized to ensure patient confidentiality. Data accuracy was verified through double-entry and periodic audits.

Statistical analysis

Descriptive statistical analysis was performed using IBM SPSS software version 25.0 (IBM Corp., Armonk, NY). Counts, proportions, averages, and standard deviations were calculated to summarize baseline characteristics and SF-12 scores. The normality of patients' SF-12 scores was assessed using the Shapiro-Wilk test. Correlation analysis included independent t-tests and one-way ANOVA to explore correlations between various sociodemographic and medical characteristics and SF-12 scores. Regression analysis encompassed linear regression to investigate the correlation between smoking pack-years and SF-12 scores, and multiple linear regression to identify independent predictors of improvements in the physical and mental components of SF-12. Statistical significance was set at P < 0.05.

## Results

Sociodemographic characteristics

The study included 275 patients. Of the patients, 59.64% were males and 40.36% were females, with an average age and standard deviation of 66.51 and 6.88, respectively. About 63% of patients had no university education, while the remaining had a university and higher education. Of the patients, 39.64% earned less than 4000 Saudi riyals (SAR), 32.73% earned between 4000 and 8000 SAR, and 27.64% earned more than 8000 SAR per month, and the majority of our patients were unemployed or retired (Table 1).

**Table 1:** Sociodemographic and medical characteristics of study participants (n = 275). Note: Categorical data are reported as frequency and percentages, while continuous data are presented as mean ± standard deviation. SAR: Saudi riyal.

Characteristic		n (%)
			Gender	Male	164 (59.64)
Female	111 (40.36)		
Age (years)		66.51 ± 6.88
Educational level	Less than high school	59 (21.46)
	High school	114 (41.46)
	Diploma	11 (4.00)
	Bachelor's	83 (30.18)
	Master's	7 (2.55)
	PhD	1 (0.36)
Income	0-4000 SAR	109 (39.64)
	4001-8000 SAR	90 (32.73)
	More than 8000 SAR	76 (27.64)
Occupational status	Unemployed/retired	209 (76)
	Employed	66 (24)
Co-morbidities	Diabetes	170 (61.18)
	Ischemic heart disease	152 (55.27)
	Dyslipidemia	198 (72)
	Hypertension	239 (86.91)
	Chronic kidney disease	46 (16.73)
	Live disease	13 (4.73)

Factors associated with quality of life

Linear regression analysis revealed a significant association between older age and lower preoperative and postoperative SF-12 scores (P = 0.025, 0.016, and <0.001, respectively). Employment correlated with increased postoperative physical and mental SF-12 scores (P < 0.001). In postoperative analysis, gender showed statistically significant differences in mental scores, with males scoring higher (P = 0.036). Lower income was associated with lower postoperative mental scores (P = 0.046). All sociodemographic characteristics exhibited significant improvements in both preoperative and postoperative physical and mental scores (Table 2).

**Table 2:** Preoperative and postoperative SF-12 scores by patient characteristics. Physical and mental component scores are presented as mean ± standard deviation. P-values are considered significant if <0.05. SF-12: Short Form Health Survey 12; SAR: Saudi riyal.

Characteristics		Preoperative SF-12 scores					Postoperative SF-12 scores				Difference between preoperative and postoperative (p-value)		
		Physical	P-value		Mental	P-value	Physical	P-value	Mental	P-value	Physical component	Mental component	
Gender	Male	54.5 ± 10.67	0.824		52.08 ± 9.67	0.979	61.24 ± 9.07	0.901	61.50 ± 7.15	0.036	<0.001	<0.001	
	Female	53.34 ± 8.50			51.98 ± 6.75		63.41 ± 7.65		58.50 ± 6.00				
Educational level	Less than high school	54.00 ± 10.0	0.754		52.80 ± 9.80	0.356	60.48 ± 7.0	0.562	58.44 ± 12.74	0.468	0.004	0.002	
	High school	56.25 ± 9.80			51.65 ± 9.00		65.06 ± 11.80		58.13 ± 11.70				
	Diploma	59.50 ± 11.50			57.20 ± 11.20		81.12 ± 10.50		74.76 ± 14.76				
	Bachelor's	50.00 ± 7.70			52.20 ± 6.20		57.60 ± 9.70		64.86 ± 8.06				
	Master's	60.70 ± 9.00			58.50 ± 9.00		69.60 ± 10.00		76.05 ± 11.70				
	PhD	54.00			52.50		60.48 ± 7.0		68.00				
Income	0-4000 SAR	55.34 ± 9.00	0.943		54.88 ± 10.00	0.612	62.87 ± 10.90	0.643	57.97 ± 10.35	0.137	0.046	0.029	
	4001-8000 SAR	51.25 ± 11.50			49.75 ± 10.50		57.37 ± 13.20		52.44 ± 10.65				
	More than 8000 SAR	53.70 ± 8.30			51.70 ± 8.30		61.07 ± 10.30		60.95 ± 10.78				
Occupational status	Unemployed/retired	51.34 ± 9.00	0.003		49.88 ± 10.00	<0.001	56.47 ± 10.80	0.006	57.37 ± 13.23	<0.001	<0.001	<0.001	
	Employed	60.70 ± 11.50			58.20 ± 11.50		67.35 ± 13.50		65.86 ± 15.00				
Co-morbidities	Diabetes	49.64 ± 9.13	<0.001	48.99 ± 8.12		0.007	56.83 ± 8.72	<0.001	57.83 ± 7.15	<0.001	<0.001	<0.001	
	Ischemic heart disease	46.19 ± 8.12	<0.001	46.5 ± 10.21		<0.001	47.43 ± 9.12	<0.001	54.43 ± 7.66	<0.001	<0.001	<0.001	
	Dyslipidemia	49.04 ± 11.23	<0.001	49.47 ± 7.77		0.018	57.39 ± 7.15	<0.001	57.39 ± 6.22	<0.001	<0.001	<0.001	
	Hypertension	51.7 ± 10.21	0.003	50.0 ± 9.85		0.046	60 ± 10.23	0.591	60.7 ± 8.9	0.591	<0.001	<0.001	
	Chronic kidney disease	47.51 ± 8.53	<0.001	50.21 ± 7.81		0.07	53.72 ± 11.51	<0.001	55.72 ± 10.01	<0.001	<0.001	<0.001	
	Live disease	47.67 ± 7.77	<0.001	48.22 ± 4.78		<0.001	53.52 ± 7.13	<0.001	54.52 ± 6.2	<0.001	<0.001	<0.001	

Significant differences were observed in preoperative SF-12 physical and mental scores based on BMI categories, with lower scores in non-normal weight patients compared to normal weight patients (P < 0.05). Lower postoperative physical and mental scores were observed in all BMI categories compared to normal weight, except for obese patients regarding postoperative mental scores. A significant correlation was observed between smoking pack-years and SF-12 change (P < 0.001, r^2^ = -0.48).

Patients with any medical comorbidity had significantly lower physical SF-12 scores compared to those without (P < 0.05). Statistically significant lower mental SF-12 scores were observed in patients with any comorbidity except for hypertension. Patients with comorbidities had lower postoperative physical and mental scores (P < 0.05). However, all mentioned comorbidities showed significant improvements in SF-12 physical and mental scores postoperatively (P < 0.001).

## Discussion

Our study aimed to assess the effectiveness of lower limb revascularization surgery on the physical and mental quality of life of patients after a two-year follow-up period. The initial observation revealed significantly low SF-12 scores in patients with peripheral arterial disease. However, all patients, regardless of sociodemographic and medical characteristics, demonstrated improvements in both their physical and mental SF-12 components postoperatively.

Male sex was identified as a predictor of better improvement in the mental component of SF-12 compared to females. This finding aligns with existing literature indicating that females often score lower on health measures compared to males [14,15]. Furthermore, unemployed or retired individuals exhibited significantly lower physical and mental scores and experienced lesser improvements in both scales, consistent with previous studies demonstrating lower long-term physical and psychological well-being in unemployed populations.

Patients' comorbidities had a substantial impact on their preoperative and postoperative physical and mental scores, with the exception of hypertension, likely due to its high prevalence among peripheral arterial disease patients. Previous research has highlighted the independent influence of comorbidities such as ischemic heart disease, liver disease, and chronic kidney disease on patients' quality of life [16]. BMI categories and smoking pack-years were significantly associated with lower physical and mental quality of life, consistent with previous studies [17]. These correlations may be attributed to their contribution to the etiopathogenesis and progression of peripheral arterial disease.

Implications of our research include the importance of targeting potential risk factors for lower improvement in the physical and mental quality of life to optimize postoperative outcomes. This includes implementing smoking cessation programs, providing weight loss advice and dietary interventions, and promoting physical activity.

The study has several limitations, including a relatively small sample size, absence of controls for disease severity, and lack of information regarding participants' psychiatric history. To overcome these limitations, future research endeavors should aim for larger-scale investigations that incorporate robust controls for confounding factors, including disease severity, and consider comprehensive assessments of participants' psychiatric history to provide a more nuanced understanding of the study outcomes.

## Conclusions

Our study aimed to identify predictors of lower quality of life following lower limb revascularization surgery in patients with peripheral arterial disease. Factors such as male sex, unemployment or retirement, and various medical comorbidities including diabetes, ischemic heart disease, dyslipidemia, chronic kidney disease, and liver disease, along with obesity and high smoking pack-years, emerged as significant predictors. These findings emphasize the importance of targeting these risk factors to optimize postoperative outcomes and enhance the physical and mental quality of life for patients undergoing revascularization surgery. Through targeted interventions, such as smoking cessation programs, weight management strategies, and comprehensive medical management, healthcare providers can strive to improve the overall well-being of patients with peripheral arterial disease undergoing surgical intervention.
